# *Ibisia marginata* (Fabricius, 1781) (Diptera, Athericidae): Distribution and Perennial Emergence Patterns in Croatia

**DOI:** 10.3390/insects16080816

**Published:** 2025-08-07

**Authors:** Marija Ivković, Jelena Fajdetić, Viktorija Ergović

**Affiliations:** 1 Division of Zoology, Department of Biology, Faculty of Science, University of Zagreb, Rooseveltov trg 6, 10000 Zagreb, Croatia; 2 Prilaz Ivana Visine 5, 10020 Zagreb, Croatia; jelenafajdetic@gmail.com; 3 Department of Biology, Josip Juraj Strossmayer University of Osijek, Cara Hadrijana 8/a, 31000 Osijek, Croatia; viktorija.ergovic@biologija.unios.hr

**Keywords:** Croatia, ecoregions, phenology, univoltine species, substrate

## Abstract

*Ibisia marginata* (Diptera, Athericidae) is an important species in macrozoobenthic communities in freshwater streams and rivers of Europe. It is a merolimnic insect whose larvae live in aquatic habitats and are predators. Pupation takes place out of water, mainly in moss, and adults live in terrestrial habitats in close proximity to water. The goals of this study were to determine the distributional patterns of *I. marginata* in Croatia, both as larvae and adults. Additionally, a goal was to recognize emergence patterns through the 16 years of research at Plitvice Lakes NP. The preference of larvae towards the different substrates represented at Plitvice Lakes NP was also analyzed. Samples of larvae and adults were collected at 50 sampling sites. Adults of *I. marginata* were collected monthly using pyramid-type emergence traps at four sampling sites in Plitvice Lakes National Park. At two of these sites, samples were collected from 2007 to 2008, while at the other two sites, sampling was conducted from 2007 to 2022. A total of 374 adult specimens were collected during the 16 years of the emergence study. *Ibisia marginata* is a univoltine species with a peak emergence in July and a flight period lasting from June to August, depending on the year. Preferred larval substrates were moss and gravel.

## Simple Summary

*Ibisia marginata* (Diptera, Athericidae) is an important predator species in macrozoobenthic communities in freshwater lotic habitats of Europe. Our study aimed to discover the wider distribution limits of *I. marginata* in Croatia and determine its perennial emergence patterns and substrate preferences at Plitvice Lakes National Park. Samples of larvae and adults were collected at 50 sampling sites. A total of 1478 larvae and 374 adult specimens were collected. Adults of *I. marginata* were collected monthly using pyramid-type emergence traps at four sampling sites in Plitvice Lakes National Park. At two of these sites, samples were collected from 2007 to 2008, while at the other two sites, sampling was conducted from 2007 to 2022. *Ibisia marginata* is a univoltine species, with a peak of emergence in July and a phenology period lasting from June to August. Preferred larval substrates are moss and gravel.

## 1. Introduction

The family Athericidae was established by Stuckenberg [1] as the group had previously been included in the family Rhagionidae. The phylogeny of the Athericidae is well resolved, and it is positioned as the sister group of the Tabanidae [2,3,4]. The family contains 13 genera [5] with more than 130 species [6], and is widely distributed in all biogeographical regions [7]. Only ten species occur in Europe [8]. Flies belonging to the Athericidae are slender, with relatively large wings that are held in a wide V-form when at rest, and some species have patterned wings. Adults feed mainly on nectar, but females of some species feed on mammalian blood [6]. The larvae are elongated, tapering anteriorly, subcylindrical, and up to about 26 mm long; they have a retractile head, eight pairs of reasonably prominent abdominal prolegs and, important for larval recognition, divergent ciliated processes on the posterior end of the abdomen. The larvae are strictly aquatic and are predators of other soft-bodied insect larvae such as Chironomidae, Tipulidae, Ephemeroptera, Plecoptera and Trichoptera [9]. They are an important ecological component in many benthic communities [10]. Studies in Europe suggest that the larvae are good indicators of substrate stability, and that pH can be limiting, with acidification having a negative effect on their occurrence. All species are univoltine; the females deposit all their eggs in a single event and then die nearby. Each female lays her eggs in a cluster, cemented to a preferred substrate that overhangs the water in which the larvae will live [9]. They prefer forest environments, or streams and rivers with dense riparian vegetation [6].

*Ibisia marginata* (Fabricius, 1781) is a widely distributed European species [10], but there are only a few records in Croatia [11]. The larval development of *I. marginata* under natural conditions was described for the first time by Vaňhara [12], and it was found that the larval development of *I. marginata* takes one year, with four instars. The presence of branches of deciduous trees hanging over the water is necessary for *I. marginata* to complete its development cycle, as females oviposit on the lower surface of the leaves of several tree species. When mature, the larvae void their gut contents, migrate out of the water, and locate sites for pupation, which always takes place out of the water and occurs frequently among bryophytes [13]. According to current knowledge, *I. marginata* is intolerant of organic pollution and prefers small, colder streams [13,14,15]. Larvae of *I. marginata* occupy aquatic sites where the water temperature in summer is not less than 11 °C. *Ibisia marginata* is a rheophilous taxon that mainly prefers streams in sub-montane and montane zones and is associated with cold streams [10]. *Ibisia marginata* prefers neutral to alkaline conditions and is sensitive to acidification [9,10].

So far, there have been no studies dealing with emergence patterns and the distribution of *I. marginata* in Croatia. The goal is to see the current distribution area of *I. marginata* and to determine its emergence patterns through the years.

## 2. Materials and Methods

### 2.1. Study Site

Croatia is a relatively small European country with a surface area of less than 57,000 km^2^. According to Illies [16], it is divided into two ecoregions, the Dinaric Western Balkan region (ER5) and the Pannonian lowland region (ER11), and forms part of two drainage basins, the Black Sea Basin and the Adriatic Sea Basin. Four sites (Tufa barrier Labudovac, Tufa barrier Kozjak–Milanovac, Tufa barrier Novakovića Brod and Village Korana) in Plitvice Lakes National Park (NP) were selected for the study of emergence patterns of *Ibisia marginata*. Plitvice Lakes NP is located in the karst region of the Dinaric Mountains in Croatia. The Plitvice Lakes barrage lake system comprises 16 mostly oligotrophic, dimictic and fluvial lakes connected by tufa barriers. The lakes are characterized by a low organic solute concentration, supersaturation with calcium salts, pH > 8.0 and the presence of algae and mosses that mediate tufa barrier formation.

### 2.2. Specimen Records

**Figure 1:**
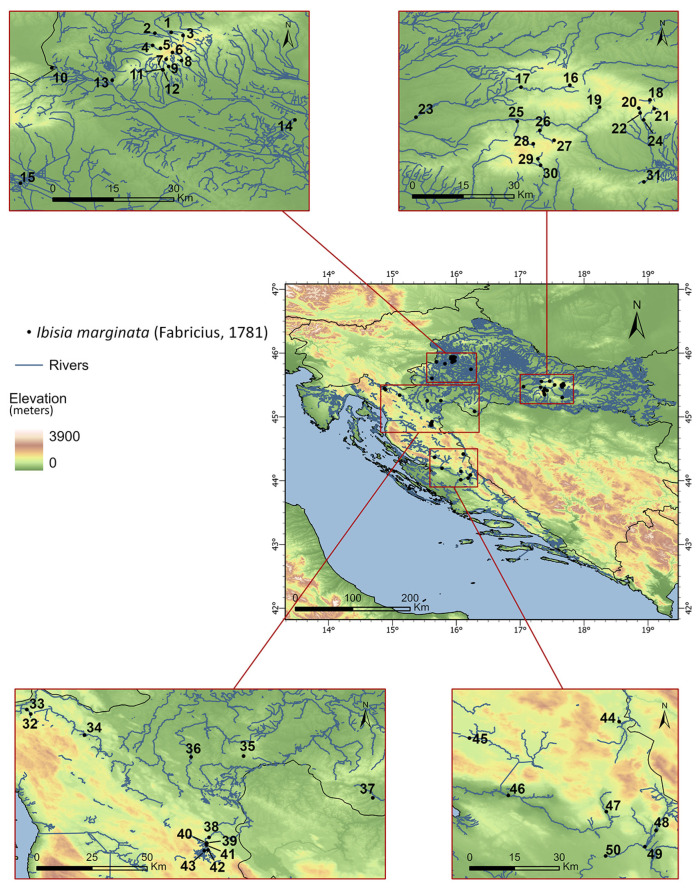
Sampling sites of *Ibisia marginata* in Croatia. Site numbers are listed in Table 1.

This paper is based on previously unpublished data obtained during monitoring and various scientific projects running over the last 18 years. Each record was georeferenced using ArcGIS Pro software (version 2.6, ESRI, Redlands, CA, USA) (Figure 1). The literature used for identification was based on Thomas [8]. Adult specimens were collected using emergence traps (details in Ivković et al. [17]), whereas larvae were collected using a Surber sampler (25 × 25 cm, 500 μm mesh size) and a kick-net sampler (25 × 25 cm, 500 μm mesh size). Larval samples were collected in the course of several macroinvertebrate surveys conducted between 2018 and 2024.

### 2.3. Sampling Procedure for Emergence Records

Pyramidal-shaped emergence traps (50 cm tall, four-sided with a 45 × 45 cm base) were placed at four sites in Plitvice Lakes NP. At Tufa barrier Novakovića Brod and Village Korana, they were only operational for two years, 2007 and 2008; at Tufa barrier Labudovac and Tufa barrier Kozjak–Milanovac, they were operational for 16 years, from 2007 to 2022. Traps were sited to guarantee a representative sampling of emergence from all the microhabitats present at each site (moss, gravel and sand). Six traps were placed at each site (two traps per substrate), attached to the streambed to allow the free movement of larvae in and out of the sampling area. The side frames of the traps were covered with 1 mm mesh netting. At the top of each trap were collecting containers filled with preservative (2% formaldehyde with a few drops of detergent). The containers were emptied at the end of each month, and samples were preserved in 80% ethanol. All the physical and chemical properties of the water at the sites can be obtained in Ivanković et al. [18].

### 2.4. Data Analysis

The Kruskal–Wallis H test (Statistica 10.0) and the Mann–Whitney U test for pairwise comparisons were performed to detect differences in the number of specimens of emerging adults among the different substrates present at the sampling sites (moss, gravel and sand) in Plitvice Lakes NP.

## 3. Results

The following format is used for the distribution data: literature references (name of the site and, in parentheses, the citation of the site ID and the reference); new records (life stage in which the identifications were made, i.e., adult ♂, ♀ and larvae; name of the site; and, in parentheses, the site ID, date of collection). All sampling sites and site numbers are listed in Table 1. This data was collected from 50 sites: 31 sites in the Pannonian lowland ecoregion and 19 sites in the Dinaric Western Balkan ecoregion. All sites are streams in a woody environment. All sites at which I. marginata was collected were streams flowing through a woody environment.

### 3.1. Literature Records of Ibisia Marginata in Croatia

Korana Village, Plitvice Lakes NP (38) [11]; Tufa barrier Novakovića Brod, Plitvice Lakes NP (39) [11]; Tufa barrier Kozjak–Milanovac, Plitvice Lakes NP (41) [11]; Tufa barrier Burget-Kozjak, Plitvice Lakes NP (42) [19]; Tufa barrier Labudovac, Plitvice Lakes NP (43) [11].

### 3.2. New Records of Ibisia Marginata in Croatia

In total, 13 larvae, Vidak, Medvednica Mountain (1), 8 July 2020; 67 larvae, same site, 8 April 2021; 18 larvae, same site, 26 October 2021; 11 larvae, Bistra, Krainje, Kraljev Vrh (2), 28 June 2022; 3 larvae, Rakova Noga, Medvednica Mountain (3), 8 July 2020; 27 larvae, same site, 8 April 2021; 21 larvae, same site, 26 October 2021; 9 larvae, Bistra lower part, Medvednica Mountain (4), 8 July 2020; 17 larvae, same site, 8 April 2021; 51 larvae, same site, 26 October 2021; 14 larvae, Bistra upper part, Medvednica Mountain (5), 8 July 2020; 18 larvae, same site, 8 April 2021; 14 larvae, same site, 26 October 2021; 1 larva, Bliznec upper part, Medvednica Mountain (6), 25 October 2021; 16 larvae, Kraljevec upper part, Medvednica Mountain (7), 9 July 2020; 42 larvae, same site, 8 April 2021; 41 larvae, same site, 25 October 2021; 12 larvae, Bliznec lower part, Medvednica Mountain (8), 9 April 2021; 16 larvae, same site, 25. October 2021; 46 larvae, Kraljevec lower part, Medvednica Mountain (9), 9 July 2020; 38 larvae, same site, 8 April 2021; 110 larvae, same site, 25 October 2021; 1 larva, Sava, Drenje–Jesenice (10), 23 July 2019; 4 larvae, Veliki Potok lower part, Medvednica Mountain (11), 10 July 2020; 38 larvae, same site, 9 April 2021; 13 larvae, same site, 25 October 2021; 6 larvae, Mali Potok lower part, Medvednica Mountain (12), 6 April 2021; 33 larvae, same site, 25 October 2021; 2 larvae, Krapina, Zaprešić (13), 29 June 2022; 1 larva, Sava, Rugvica (14), 23 July 2019; 1 larva, Kupčina, Lazina (15), 28 June 2024; 102 larvae, Bijela upper part, Papuk Mountain (16), 31 July 2020; 1 larva, same site, 29 April 2021; 25 larvae, same site, 21 October 2021; 3 larvae, Bijela lower part, Papuk Mountain (17), 21 October 2021; 17 larvae, Kovačica upper part, Papuk Mountain (18), 30 July 2020; 73 larvae, same site, 16 April 2021; 67 larvae, same site, 19 October 2021; 9 larvae, Brzaja upper part, Papuk Mountain (19), 30 July 2020; 2 larvae, same site, 15 April 2021; 4 larvae, same site, 21 October 2021; 17 larvae, Veličanka upper part, Papuk Mountain (20), 16 April 2021; 7 larvae, same site, 19 October 2021; 9 larvae, Dubočanka upper part, Papuk Mountain (21), 1 August 2020; 117 larvae, same site, 16 April 2021; 43 larvae, same site, 19 October 2021; 25 larvae, Veličanka lower part, Papuk Mountain (22), 30 July 2020; 9 larvae, same site, 16 April 2021; 14 larvae, same site, 19 October 2021; 2 larvae, same site, 16 April 2021; 6 larvae, same site, 19 October 2021; 1 larva, Bijela Rijeka, road Gaj–Parmakovac (23), 14 May 2021; 5 larvae Dubočanka lower part, Papuk Mountain (24), 1 August 2020; 1 larva, Sivornica lower part, Psunj Mountain (25), 31 July 2020; 6 larvae, same site, 15 April 2021; 6 larvae, Cikotska lower part, Psunj Mountain (26), 31 July 2020; 10 larvae, same site, 15 April 2021; 19 larvae, same site, 23 October 2021; 37 larvae, Cikotska upper part, Psunj Mountain (27), 23 October 2021; 29 larvae, Sivornica upper part, Psunj Mountain (28), 31 July 2020; 43 larvae, same site, 15 April 2021; 11 larvae, same site, 23 October 2021; 4 larvae, Šumetlica, above Šibnjak (29), 8 June 2020; 4 larvae, Šumetlica upper part, Psunj Mountain (30), 24 August 2013; 4 larvae, Vučjak (31), 24 August 2023; 7 larvae, Curak, after HE Munjara (32), 28 June 2018; 2 larvae, Curak, Donji Ložac (33), 28 June 2018; 2 larvae, Ribnjak, before mouth to Dobra River (34), 22 July 2019; 2 larvae, Brusovača, Sagradžije (35), 29 August 2023; 1 larva, Korana, Veljun (36), 4 September 2024; 1 larva, Ljubina, Donja Ljubina (37), 8 June 2020; 2♀, 3♂, Korana Village, Plitvice Lakes NP (38), 29 June 2007; 1♂, same site, 25 July 2007; 9♀, 3♂, same site, 26 July 2008; 1♀, 2♂, same site, 29 August 2008; 3♀, Tufa barrier Novakovića Brod, Plitvice Lakes NP (39), 29 June 2007; 37♀, 31♂, same site, 25 July 2007; 7♀, 1♂, same site, 30 August 2007; 1♂, same site, 29 June 2008; 7♀, 3♂, same site, 26 July 2008; 6♀, 5♂, same site, 29 August 2008; 1♀, Stream Plitvica, Plitvice Lakes NP (40), 25 July 2008; 1♀, 1♂, Tufa barrier Kozjak–Milanovac, Plitvice Lakes NP (41), 27 July 2017; 1♂, same site, 31 July 2018; 1♂, same site, 26 July 2019; 4♀, 1♂, same site, 30 June 2020; 1♀, same site, 31 July 2020; 1♀, 1♂, same site, 30 June 2021; 1♀, 6♂, same site, 30 July 2021; 1♀, 1♂, same site, 31 August 2021; 1♀, 1♂, same site, 28 July 2022; 1♂, Tufa barrier Labudovac, Plitvice Lakes NP (43), 25 July 2007; 1♂, same site, 30 June 2008; 4♀, 2♂, same site, 31 July 2011; 6♀, 5♂, same site, 31 July 2013; 6♀, 10♂, same site, 25 July 2014; 2♀, same site, 31 August 2014; 34♀, 25♂, same site, 24 July 2015; 11♀, 7♂, same site, 25 July 2016; 2♀, same site, 31 August 2016; 3♂, same site, 27 June 2017; 9♀, 16♂, same site, 27 July 2017; 1♀, same site, 29 August 2017; 2♀, 4♂, same site, 31 July 2018; 2♀, 1♂, same site, 26 July 2019; 8♀, 5♂, same site, 31 July 2020; 3♀, 3♂, same site, 30 July 2021; 1♀, same site, 31 August 2021; 3♀, 2♂, same site, 28 July 2022; 1 larva, Joševica, bridge on a road D. Suvaja–Brotnja (44), 8 April 2024; 1 larva, Opsenica, Jurjević (45), 2 October 2023; 2 larvae, Zrmanja, Berberov Buk (46), 2 October 2023; 1 larva, same site, 8 April 2024; 1 larva, Zrmanja, Palanka (47), 9 August 2019; 1 larva, Drain ditch HE Golubić, before the mouth to Butižnica (48), 3 May 2019; 8 larvae, Butižnica, Bulin Most (49), 6 March 2024; 3 larvae, Bilušića Buk, Krka (50), 26 August 2024.

### 3.3. Ibisia Marginata Sex Ratio, Emergence Patterns and Microhabitat Preference

During our study of emergence patterns in Plitvice Lakes NP from 2007 until 2022, 374 specimens of *Ibisia marginata* were collected. Males of *I. marginata* were more abundant at the Tufa barrier Labudovac in 2014, 2017 and 2018 (55.5%, 65.5% and 66.6%, respectively), at the Tufa barrier Kozjak–Milanovac in 2020 (66.6%), and at Korana Village in 2007 (66.6%). Females were more abundant at the Tufa barrier Labudovac in 2011, 2015, 2016 and 2020 (66.6%, 57.6%, 65.0% and 51.5%, respectively), at the Tufa barrier Kozjak–Milanovac in 2021 (72.7%), at the Tufa barrier Novakovića Brod in 2007 and 2008 (62.1% and 59.0%, respectively), and at Korana Village in 2008 (72.7%) (Table 2). Emergence started in June 2007 and 2008, with four and five specimens collected at Tufa barrier Novakovića Brod and Village Korana in 2007 and 2008, respectively, and one specimen collected in June at Tufa barrier Labudovac in 2017. Emergence lasted during August only in 2017 and 2021, with one specimen collected at Tufa barrier Labudovac in both years. All other specimens collected emerged in July at all studied sites.

**Figure 2:**
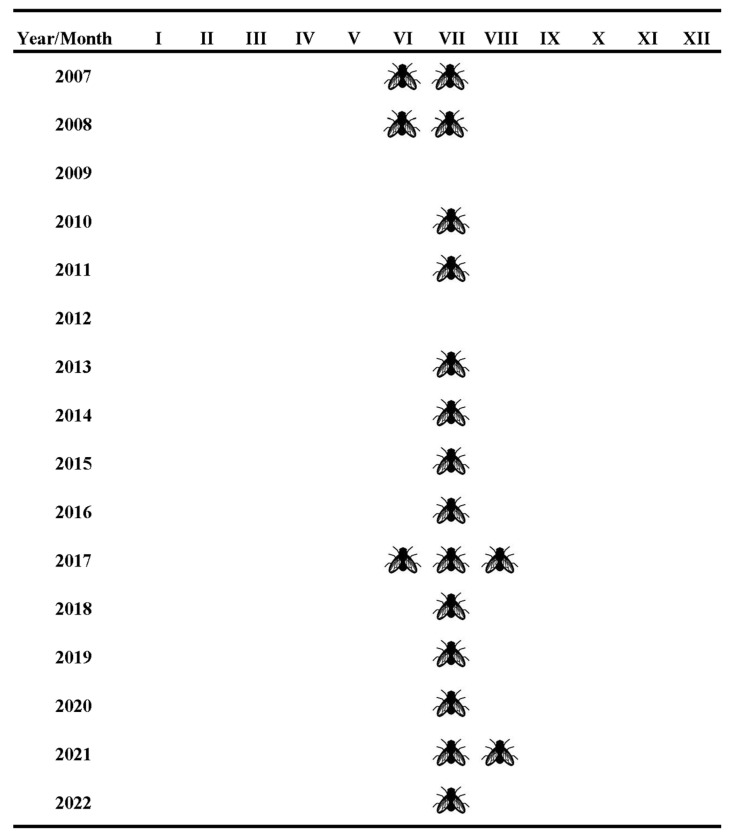
*Ibisia marginata* flight period in emergence traps at Plitvice Lakes National Park during 16-year period (2007–2022).

*Ibisia marginata* had one generation per year according to emergence data. The peak emergence was in July, but the emergence period was throughout the summer months, from June to August (Figure 2).

There is a statistically significant difference for *I. marginata* between substrate types. Moss and gravel substrates were significantly greater than sand substrates (H = 12.553, df = 2, N = 172, *p* = 0.0019). This indicates that larval *I. marginata* prefers these substrate types for pupation.

## 4. Discussion

*Ibisia marginata* was recorded in Croatia for the first time by Ivković et al. [11], at several sites in Plitvice Lakes NP. In this study, the distribution of the species is revealed to be much more extensive in Croatia, and it is present both in the Dinaric Western Balkan (ER5) and Pannonian lowland (ER11) ecoregions [16]. There are no records for Croatia in GBIF.org [20], but there are many records from other European countries.

During the 16 years of research into the emergence of *I. marginata*, it was established that it is most likely a univoltine species with an emergence peak in July, as previously noted by Samietz [21]. However, in our study, emergence started earlier than previously noted, with some specimens emerging during June. This could be because of the higher water temperatures in those years, as higher temperatures have an influence on the beginning of emergence, as noted in other Diptera families in Plitvice Lakes NP [22,23,24,25,26]. Although in some years there were more males or more females caught in the traps, there are no studies that deal with changes in the sex ratio of *I. marginata*. Since the flight period is relatively short (about 6 weeks) [21], there is probably no great difference in the beginning of emergence between males and females. However, the imago only lives up to 10 days [12], and since our samples were collected on a monthly basis and not daily, we cannot really state that with certainty. *Ibisia marginata* occurs in clean upland areas and prefers colder and calcareous streams with sufficient flow [14], and this is the case with all the sites where the species occurred in this research. Larvae of *Ibisia marginata* prefer moss and gravel [24] as the substrate from which the adults emerge, and this was not surprising. Similar results were obtained for the family Athericidae (not identified to species level) from Plitvice Lakes NP by Čmrlec et al. [24], and it is known that the larvae pupate mainly on a moss substrate [8].

## 5. Conclusions

*Ibisia marginata* is widely distributed in Croatia, occurring in streams in woody environments in the Dinaric Western Balkan (ER5) and Pannonian lowland (ER11) ecoregions. It has a short flight period that is confined to summer months, with peak emergence in July. Larvae prefer to pupate on moss and gravel substrates. As *Ibisia marginata* is an important predator in small, clean streams of woody environments, research into its ecological preference and its distribution patterns is of great significance.

## Figures and Tables

**Table 1:** Sampling sites in Croatia. Ecoregions are taken from Illies [16], Dinaric Western Balkan (5) and Pannonian lowland (11).

Site ID	Site Name	Latitude N	Longitude E	Ecoregion
1	Vidak, Medvednica Mountain	45.94183936	15.95409444	11
2	Bistra, Krainje, Kraljev Vrh	45.93993024	15.91781770	11
3	Rakova Noga, Medvednica Mountain	45.93487495	15.98044463	11
4	Bistra lower part, Medvednica Mountain	45.91271993	15.91256564	11
5	Bistra upper part, Medvednica Mountain	45.90622169	15.93040591	11
6	Bliznec upper part, Medvednica Mountain	45.89693777	15.95708916	11
7	Kraljevec upper part, Medvednica Mountain	45.88191829	15.94271976	11
8	Bliznec lower part, Medvednica Mountain	45.87883161	15.97694553	11
9	Kraljevec lower part, Medvednica Mountain	45.86568890	15.94832020	11
10	Sava, Drenje–Jesenice	45.86245210	15.68805809	11
11	Veliki Potok lower part, Medvednica Mountain	45.85826568	15.93493872	11
12	Mali Potok lower part, Medvednica Mountain	45.85792332	15.93607945	11
13	Krapina, Zaprešić	45.83477084	15.82281000	11
14	Sava, Rugvica	45.74637011	16.22970883	11
15	Kupčina, Lazina	45.60521893	15.61821747	11
16	Bijela upper part, Papuk Mountain	45.56044296	17.46119028	11
17	Bijela lower part, Papuk Mountain	45.55547138	17.33138752	11
18	Kovačica upper part, Papuk Mountain	45.52116723	17.67385288	11
19	Brzaja upper part, Papuk Mountain	45.50234390	17.53991314	11
20	Veličanka upper part, Papuk Mountain	45.49970072	17.64442020	11
21	Dubočanka upper part, Papuk Mountain	45.49806672	17.68511005	11
22	Veličanka lower part, Papuk Mountain	45.48667211	17.64768756	11
23	Bijela Rijeka, road Gaj–Parmakovac	45.47522567	17.05255201	11
24	Dubočanka lower part, Papuk Mountain	45.46807177	17.65713968	11
25	Sivornica lower part, Psunj Mountain	45.46456513	17.32180235	11
26	Cikotska lower part, Psunj Mountain	45.44006793	17.38145505	11
27	Cikotska upper part, Psunj Mountain	45.41408215	17.41907392	11
28	Sivornica upper part, Psunj Mountain	45.40483059	17.36398750	11
29	Šumetlica, above Šibnjak	45.36448893	17.37629850	11
30	Šumetlica upper part, Psunj Mountain	45.34733854	17.38316746	11
31	Vučjak	45.30322214	17.65763492	11
32	Curak, after HE Munjara	45.42725431	14.89289585	5
33	Curak, Donji Ložac	45.44563232	14.87656059	5
34	Ribnjak, before mouth to Dobra River	45.34131922	15.11194526	5
35	Brusovača, Sagradžije	45.25560082	15.75924624	5
36	Korana, Veljun	45.25251358	15.54573548	5
37	Ljubina, Donja Ljubina	45.08649749	16.28549354	5
38	Korana Village, Plitvice Lakes NP	44.92583330	15.61916667	5
39	Tufa barrier Novakovića Brod, Plitvice Lakes NP	44.90222220	15.61055556	5
40	Stream Plitvica, Plitvice Lakes NP	44.90222220	15.60750000	5
41	Tufa barrier Kozjak–Milanovac, Plitvice Lakes NP	44.89416670	15.60888889	5
42	Tufa barrier Burget-Kozjak, Plitvice Lakes NP	44.87416670	15.61472222	5
43	Tufa barrier Labudovac, Plitvice Lakes NP	44.87138890	15.59972222	5
44	Joševica, bridge on road D. Suvaja–Brotnja	44.41706228	16.10935161	5
45	Opsenica, Jurjević	44.36776866	15.65878303	5
46	Zrmanja, Berberov Buk	44.19550330	15.77585642	5
47	Zrmanja, Palanka	44.14689633	16.07108783	5
48	Drain ditch HE Golubić, before the mouth to Butižnica	44.08979170	16.22053196	5
49	Butižnica, Bulin Most	44.04092978	16.18693474	5
50	Bilušića Buk, Krka	44.01308610	16.06867778	5

**Table 2:** Abundances of *Ibisia marginata* at Plitvice Lakes National Park during the 16-year study period. BL–Tufa barrier Labudovac; BKM–Tufa barrier Kozjak–Milanovac; BNB–Tufa barrier Novakovića brod; KS–Village Korana.

Site	Year	*Ibisia marginata* (Fabricius, 1781)		
		♂	♀	∑
BL	2007	1	0	1
	2008	1	0	1
	2009	0	0	0
	2010	1	0	1
	2011	2	4	6
	2012	0	0	0
	2013	5	6	11
	2014	10	8	18
	2015	25	34	59
	2016	7	13	20
	2017	19	10	29
	2018	4	2	6
	2019	1	2	3
	2020	5	8	13
	2021	3	4	7
	2022	2	3	5
BKM	2007	0	0	0
	2008	0	0	0
	2009	0	0	0
	2010	0	0	0
	2011	0	0	0
	2012	0	1	1
	2013	0	0	0
	2014	0	1	1
	2015	0	0	0
	2016	0	0	0
	2017	1	1	2
	2018	1	0	1
	2019	1	0	1
	2020	4	2	6
	2021	3	8	11
	2022	1	1	2
BNB	2007	45	74	119
	2008	9	13	22
KS	2007	4	2	6
	2008	6	16	22

## Data Availability

Data supporting the reported results can be provided upon contacting the corresponding author.
